# Antigenic Variation in *Streptococcus pneumoniae* PspC Promotes Immune Escape in the Presence of Variant-Specific Immunity

**DOI:** 10.1128/mBio.00264-18

**Published:** 2018-03-13

**Authors:** M. Georgieva, L. Kagedan, Ying-Jie Lu, C. M. Thompson, M. Lipsitch

**Affiliations:** a Center for Communicable Disease Dynamics, Department of Epidemiology, Harvard T.H. Chan School of Public Health, Boston, Massachusetts, USA; b Department of Immunology and Infectious Diseases, Harvard T.H. Chan School of Public Health, Boston, Massachusetts, USA; c Division of Infectious Diseases, Boston Children's Hospital, Harvard Medical School, Boston, Massachusetts, USA; University of Mississippi Medical Center

**Keywords:** PspC, *Streptococcus pneumoniae*, antigenic variation

## Abstract

Genomic analysis reveals extensive sequence variation and hot spots of recombination in surface proteins of *Streptococcus pneumoniae*. While this phenomenon is commonly attributed to diversifying selection by host immune responses, there is little mechanistic evidence for the hypothesis that diversification of surface protein antigens produces an immune escape benefit during infection with *S. pneumoniae*. Here, we investigate the biological significance of sequence variation within the *S. pneumoniae* cell wall-associated pneumococcal surface protein C (PspC) protein antigen. Using *pspC* allelic diversity observed in a large pneumococcal collection, we produced variant-specific protein constructs that span the sequence variability within the *pspC* locus. We show that antibodies raised against these PspC constructs are variant specific and prevent association between PspC and the complement pathway mediator, human factor H. We found that PspC variants differ in their capacity to bind factor H, suggesting that sequence variation within *pspC* reflects differences in biological function. Finally, in an antibody-dependent opsonophagocytic assay, *S. pneumoniae* expressing a PspC variant matching the antibody specificity was killed efficiently. In contrast, killing efficacy was not evident against *S. pneumoniae* expressing mismatched PspC variants. Our data suggest that antigenic variation within the PspC antigen promotes immune evasion and could confer a fitness benefit during infection.

## IMPORTANCE

Loci encoding surface protein antigens in *Streptococcus pneumoniae* are highly polymorphic. It has become a truism that these polymorphisms are the outcome of selective pressure on *S. pneumoniae* to escape host immunity. However, there is little mechanistic evidence to support the hypothesis that diversifying protein antigens produces a benefit for the bacteria. Using the highly diverse *pspC* locus, we have now characterized the functional and immune implications of sequence diversity within the PspC protein. We have characterized the spectrum of biological function among diverse PspC variants and show that *pspC* sequence diversity reflects functional differences. Further, we show that sequence variation in PspC confers an immune escape benefit in the presence of anti-PspC variant-specific immunity. Overall, the results of our studies provide insights into the functional implications of protein sequence diversity and the role of variant-specific immunity in its maintenance.

## INTRODUCTION

The bacterium *Streptococcus pneumoniae* (pneumococcus) is a respiratory commensal and pathogen characterized by extensive sequence variation. Diversity within the pneumococcal capsule is well characterized and central to the design of pneumococcal vaccines (1). Extensive research on capsular diversity has contributed greatly to our understanding of its biological importance and the critical role of antibody-mediated immunity in its maintenance (2, 3). Moreover, genetic diversity in *S. pneumoniae* extends beyond the capsule locus. Loci encoding surface protein antigens are also highly variable and subject to frequent recombination events (4, 5). Despite a growing body of evidence pointing to extensive sequence variation in pneumococcal surface protein antigens, we know little about the immune and functional implications of this diversity.

Pneumococcal surface protein C (PspC; also known as CbpA, Hic, and SpsA) is a cell wall-associated surface protein known to interact with three different human proteins via its α-helical domain (6). PspC binds the secretory component of immunoglobulin A (sIgA), complement component C3, and complement factor H (7–9). Mutants of *S. pneumoniae* that do not express PspC showed reduced virulence in mouse models of bacteremia, pneumonia, and nasal colonization (10–12). While the functional mechanisms by which PspC contributes to virulence are incompletely characterized, interactions between PspC and host proteins seem to be critical in this context. It has been proposed that the PspC-sIgA interaction facilitates adherence and subsequent invasion via the polymeric Ig receptor on mucosal epithelial cells (13, 14). Additionally, the association between PspC and the fH and C3 complement mediators seems to play an important role at various stages of the *in vivo* biology of the pneumococcus (15–22).

PspC is highly immunogenic, and anti-PspC antibodies are a major component of antibody immunity against *S. pneumoniae* (23–29). Several papers have demonstrated the protective capacity of anti-PspC immunity against carriage (10) or invasive challenge (6, 30) in mice.

Although the *pspC* gene is present in almost all pneumococci, it is highly polymorphic, and pneumococcal strains differ greatly in the particular antigenic variants that they express (6, 31). Like other surface antigens, PspC is commonly assumed to be under the control of diversifying selection to escape antibody action. Two lines of evidence support this reasoning. First, there is evidence for a concentration of nonsynonymous substitutions within the epitope regions of pneumococcal protein antigens (32). Second, there is age-specific variation in gene content, such that common variants of some core surface proteins, as well as surface proteins that are part of the accessory genome, are more common among isolates from younger (presumably immunologically naive) carriers of *S. pneumoniae* (5, 33). Relatedly, we recently reported that individuals with strong antibody responses to a particular variant of one protein antigen, pneumococcal surface protein C (PspC), are more likely, if carrying a pneumococcus, to carry one expressing a different PspC variant (23). Such results support the conjecture that common variants are net-beneficial in younger/more naive hosts but are subject to immune responses that select against their presence in more immunologically experienced people.

Despite the sequence evidence suggesting diversifying selection, mechanistic studies designed to assess the degree of allelic specificity of anti-PspC antibody responses have had heterogeneous results. While such variation probably partly reflects real variability in the extent of activity by antibodies raised to one allele against other alleles, it may also reflect variability due to strain background (21, 34) as well as cross-reactions with the paralogous PspA protein (6). A study of antibodies to three recombinant PspC immunogens showed a range in the extent of specificity, with antibodies to a PspC of group 8 binding by Western blotting and flow cytometry only to a strain containing that allele, anti-PspC3 antibodies showing binding to multiple PspC alleles (but narrower specificity in functional activities such as blocking of PspC interactions with host proteins), and anti-PspC4 antibodies showing binding also to PspA (35). In another study, opsonic and opsonophagocytic killing activity of anti-recombinant PspC antibodies was limited to the isolate from which the recombinant *pspC* gene was obtained and was not observed with two other strains (36).

Given these differing results and the potential for the strain background to affect PspC activity and the extent of antibody action against PspC, we sought to study PspC diversity using isogenic strains that differ only at the *pspC* locus. Within a data set of 616 asymptomatically carried *S. pneumoniae* isolates, we identified four PspC variants that span the sequence diversity within the *pspC* locus (the sequences are included in Text S1) (5). Here, we show that sequence variability within PspC, in an otherwise fixed genetic background, can create differences in the ability of strains to bind human factor H and can promote immune escape in opsonophagocytic assays in the presence of variant-specific immunity. Our results provide insight into the biological implications of PspC sequence diversity and the role of variant-specific antibodies in antipneumococcus immunity.

10.1128/mBio.00264-18.1 TEXT S1  Sequences of full-length PspC variants and truncated protein fragments (in gray). Download TEXT S1, PDF file, 0.1 MB. Copyright © 2018 Georgieva et al. 2018 Georgieva et al. This content is distributed under the terms of the Creative Commons Attribution 4.0 International license.

## RESULTS

### Purification of recombinant PspC protein variant fragments and production of variant-specific antibodies.

**FIG 1:**
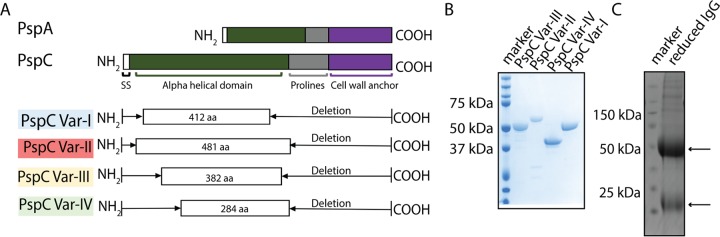
Purification of recombinant PspC protein variants and production of variant-specific antibodies. (A) Four variant-specific recombinant protein constructs were designed to capture the sequence variability within the PspC protein. Annotated are the N-terminal signal sequence (SS), the alpha helical domain, the proline-rich domain, and the choline-rich cell wall anchor region within PspC. The truncated PspC protein constructs excluded epitopes conserved among the four variants as well as regions homologous with PspA. aa, amino acids. (B) The purity of the produced proteins was checked by SDS-PAGE analysis. (C) SDS-PAGE of IgG isolated from variant-specific rabbit antisera. The arrows indicate the 50-kDa and 25-kDa molecular mass (MM) bands seen under reducing conditions, corresponding to the IgG heavy and light chains, respectively.

Previous analysis of whole-genome sequence data from 616 asymptomatically carried *S. pneumoniae* isolates showed extensive sequence variation and recombination hot spots in the *S. pneumoniae* PspC surface protein (5, 23). For our studies here, we selected four PspC variants that span the range of genetic diversity of the *pspC* locus within this pneumococcal population (5, 23, 31). We recently reported the presence of naturally acquired antibodies against these four PspC variants in individuals colonized with *S. pneumoniae* (23). In order to begin mechanistic studies, we first generated recombinant variant-specific PspC protein constructs (see Fig. S3 in the supplemental material). In order to capture the unique sequence characteristics of each variant, we designed truncated PspC fragments which exclude conserved epitopes common among the variants as well as any epitopes shared with PspA, another highly immunogenic pneumococcal surface protein with homology to PspC (Fig. 1A). We then cloned each of these constructs into expression vectors, thereby generating PspC expression constructs bearing a C-terminal polyhistidine tag. Each PspC variant fragment was expressed in *Escherichia coli* and isolated from the protein-soluble fraction using a nickel-based on-column purification strategy. The protein-rich elution fraction was further purified on a size exclusion column, and the purity of proteins was confirmed by SDS-PAGE (Fig. 1B). For production of anti-PspC variant-fragment-specific antibodies, we immunized individual rabbits with each of the recombinant PspC variant fragments. To isolate serum immunoglobulin G (IgG) with affinity for the PspC protein while also minimizing the presence of nonspecific IgG, we first preabsorbed serum samples with lysates of ΔPspC *S. pneumoniae* and then purified the total IgG fraction as described in Materials and Methods (Fig. 1C).

### Anti-PspC antibodies are specific for their cognate PspC variant.

**FIG 2:**
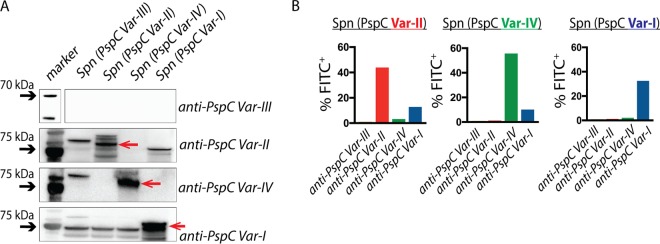
Anti-PspC antibodies are variant specific. (A) Western blot analysis of reactivity of anti-PspC var.-I, anti-PspC var.-II, anti-PspC var.-III, and anti-PspC var.-IV serum samples to whole-cell lysates of pneumococcal strains expressing different full-length PspC variants (shown at the top of the panel). Each variant-specific protein band is marked with an arrow. (B) Flow cytometry analysis for binding of variant-specific IgG to isogenic *S. pneumoniae* strains expressing a unique PspC variant. Using FITC^+^ conjugated anti-rabbit IgG secondary antibody, we evaluated binding of anti-PspC IgG to whole *S. pneumoniae* strains. The total percentage of FITC^+^ cells is shown for each *S. pneumoniae* strain incubated separately with anti-PspC var.-I, anti-PspC var.-II, anti-PspC var.-III, or anti-PspC var.-IV.

To determine the specificity of IgG for each PspC variant, we performed Western blotting of whole-cell lysates of *S. pneumoniae* strains expressing one of the four PspC variants (the full protein and not the fragment used for immunization). As shown in Fig. 2A, anti-PspC variant I (var.-I), anti-PspC var.-II, and anti-PspC var.-IV recognized with specificity the cognate PspC variant. In contrast, anti-PspC var.-III antibody did not recognize any PspC variant, suggesting that PspC var.-III recombinant protein might have been poorly immunogenic in rabbits or that anti-PspC var.-III IgG may have low affinity for its target. To determine whether these anti-PspC antibodies bind their cognate targets in the context of whole *S. pneumoniae* bacteria, we performed flow cytometry analysis of anti-PspC IgG binding to isogenic, whole *S. pneumoniae* bacteria that differed solely in the PspC variant that they express. Using a fluorescein isothiocyanate-positive (FITC^+^) conjugated anti-rabbit secondary antibody, we measured the percentages of bacterial cells with surface-deposited anti-PspC IgG (Fig. 2B). Consistent with the Western blot data, anti-PspC var.-I, anti-PspC var.-II, and anti-PspC var.-IV antibodies were specific for their cognate PspC variant in intact bacteria. Graphs show the percentages of the total bacterial population that were FITC^+^ for bound IgG. The total proportion of FITC^+^ cells was below 100% because some proportion of the cells remained unstained.

### Anti-PspC antibodies prevent interaction between PspC and fH but not between PspC and sIgA.

**FIG 3:**
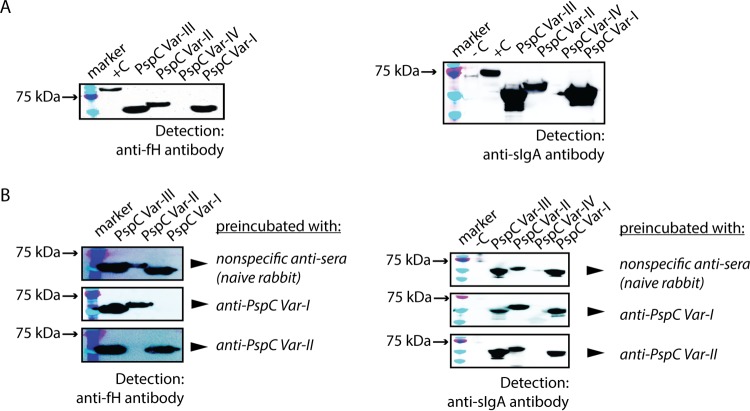
Anti-PspC IgG prevents association between PspC and the complement pathway mediator fH but not secretory IgA. (A) Western blot analysis of fH and sIgA binding to recombinant PspC proteins (truncated versions). Recombinant PspC proteins were separated by the use of 10% SDS-PAGE and transferred onto a nitrocellulose membrane, and the membrane was then incubated with recombinant human factor H protein prior to addition of anti-factor H antibody to detect fH binding. For sIgA binding, recombinant sIgA was added followed by anti-sIgA antibody. The positive control for fH detection included recombinant fH protein (+C lane). The positive control for sIgA detection included recombinant sIgA (+C lane). (B) Recombinant PspC proteins were first incubated with their respective variant-specific anti-PspC antibody followed by incubation with recombinant human factor H or sIgA. Detection was performed using anti-fH or anti-sIgA antibody. Antibody isolated from nonimmunized rabbit serum was used as a negative control. Pneumolysin pneumococcal protein (53 kDa) was included as a negative control (-C lane).

Several previous studies showed the interactions between PspC and the complement pathway mediator fH and secretory IgA (7, 8, 14, 15, 17, 19, 20, 22, 35, 37–43). Because those studies used laboratory strains of *S. pneumoniae* and clinical isolates bearing PspC variants different from those included here, we wanted to investigate whether each of the four PspC variants in our study interacted similarly with fH and sIgA. To look at PspC interaction with fH and sIgA, we performed Far-Western blotting. Recombinant PspC variant fragments were separated by SDS-PAGE, transferred onto a nitrocellulose membrane, and then incubated with recombinant fH or sIgA. Detection performed with fH-specific or sIgA-specific antibodies showed evidence for interactions among PspC var.-I, var.-II, var.-III, and fH/sIgA (Fig. 3A). In contrast, truncated PspC var.-IV did not bind fH or sIgA. Overall, our data on the interaction of PspC var.-I, var.-II, and var.-III with fH or sIgA are consistent with those from other studies. Our findings also suggest that, despite extensive sequence variability within the *pspC* locus, these biological interactions are likely important *in vivo* and remain preserved across a wide range of antigenic variants.

Previous studies on PspC and on fH and sIgA have also shown the role of these interactions in promoting immune evasion and epithelial adhesion during infection with *S. pneumoniae* (7, 14, 17, 19, 40, 44). Furthermore, a few studies have demonstrated that the fH/sIgA binding regions on PspC are targets for anti-PspC antibodies during infection and that these anti-PspC antibodies have the capacity to impede the interaction between PspC and fH/sIgA (35, 36). However, to our knowledge, in one of these studies it remained unclear whether these antibodies function in a variant-specific manner to block these interactions (36). In another study, antibodies raised against different PspC variants showed a range of specificities against pneumococcal protein lysates (35). These results showing various specificities are likely explained by the utilization of full-length PspC proteins to generate antibodies. In our study, truncated protein PspC variants (representing the unique portion of the PspC sequence) were used to generate anti-PspC IgG, allowing us to ask questions about antibody specificity while reducing the extent of cross-reactivity between PspC variants and between PspC and PspA. We separated our recombinant PspC variants by SDS-PAGE, transferred the protein sample onto a nitrocellulose membrane, and then incubated with anti-PspC antibody prior to addition of recombinant fH or sIgA. We found that anti-PspC var.-I and var.-II antibodies prevented association between recombinant PspC and fH in a variant-specific fashion (Fig. 3B). In contrast, association between PspC and sIgA remained unaffected in the presence of these antibodies, consistent with previous findings showing that fH and sIgA have distinct binding domains within PspC (38). Note that we found differences in the specificities of IgGs isolated from different rabbit antisera for PspC var.-II. A set of anti-PspC var.-II IgG isolates collected from a second rabbit was cross-reactive with PspC var.-III (Fig. S2). However, we obtained consistent results for var.-I by the use of antisera from two different rabbits.

### PspC variants differ in their capacity to bind human fH.

**FIG 4:**
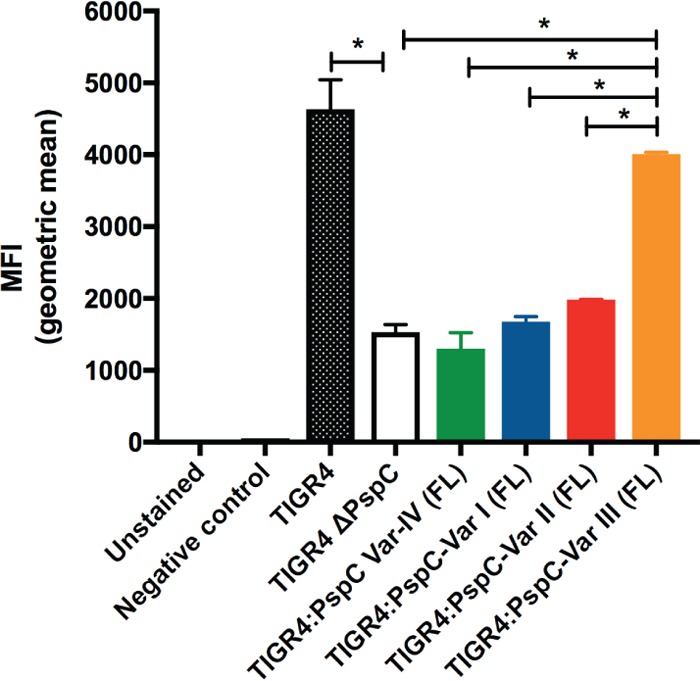
PspC variants differ in their capacity to bind fH. Flow cytometry analysis of fH binding to *S. pneumoniae* strains incubated with normal human serum as a source of fH was performed. Pneumococci with bound fH had increased levels of mean fluorescence intensity (MFI) compared to background fluorescence (negative control). We tested TIGR4 isogenic strains that express a unique full-length PspC variant. FL, full-length. *, *P* < 0.01.

To investigate how sequence variation within PspC may impact biological function, we compared the different capacities of the PspC protein variants to bind fH. We generated TIGR4 isogenic strains expressing a unique full-length PspC variant. We incubated each *S. pneumoniae* strain with IgG-depleted normal human serum as a source of factor H and measured the deposition of fH on the bacterial surface by flow cytometry as described in Materials and Methods. TIGR4 expressing var.-III bound significantly more fH (Fig. 4); in contrast, TIGR4 expressing var.-I, var.-II, or var.-IV PspC bound significantly lower levels of fH. Under these conditions, the original *S. pneumoniae* PspC var.-III strain similarly bound more fH than the *S. pneumoniae* strains bearing var.-II or var.-I. Overall, these data indicate that sequence variation within PspC encodes functional differences between antigenic variants.

### Sequence diversity within *pspC* promotes immune escape in the presence of variant-specific immunity.

**FIG 5:**
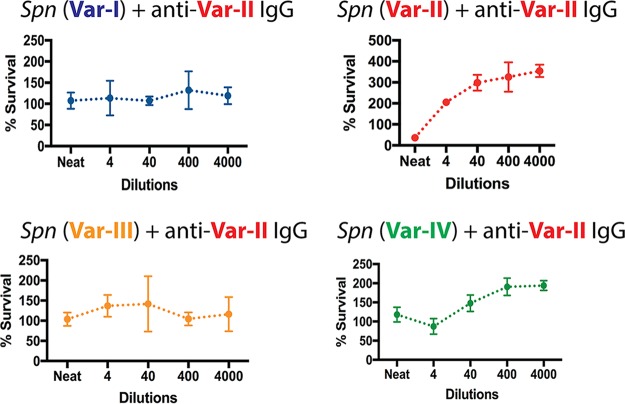
Antibody-dependent opsonophagocytic killing in the presence of anti-PspC IgG and neutrophil-like HL-60 cells. Graphs show percent survival of isogenic *S. pneumoniae* strains expressing one of the four PspC variants incubated with anti-PspC var.-II IgG. To determine the percentage of CFU that survived under each condition, CFU counts at the assay end time point were divided by the CFU counts at time point 0.

To measure the impact of variant-specific immunity against PspC on bacterial survival, we performed antibody-dependent opsonophagocytic killing assays. We found that *S. pneumoniae* expressing PspC var.-II was killed efficiently in the presence of anti-PspC var.-II antibody. In contrast, killing efficacy was not evident against *S. pneumoniae* expressing a mismatched PspC variant (Fig. 5). Within our set of anti-PspC antibodies and under the conditions tested here, only anti-PspC var.-II was opsonic whereas anti-PspC var.-I and anti-PspC var.-IV were nonopsonic (Fig. S1). While anti-PspC var.-I IgG and anti-PspC var.-IV IgG are specific for their target as shown by Western blotting and flow cytometry (Fig. 2), they seem nonfunctional in opsonophagocytic assays.

10.1128/mBio.00264-18.2 FIG S1  Anti-PspC var.-I IgG (A) and var.-IV IgG (B) are nonopsonic in a neutrophil-mediated antibody-dependent opsonophagocytic assay. Graphs show percent survival of isogenic *S. pneumoniae* strains incubated with anti-PspC var.-I IgG or var.-IV IgG. Download FIG S1, PDF file, 0.4 MB. Copyright © 2018 Georgieva et al. 2018 Georgieva et al. This content is distributed under the terms of the Creative Commons Attribution 4.0 International license.

10.1128/mBio.00264-18.3 FIG S2  Variability in anti-PspC var.-II IgG specificity depending on the source of antisera. (A) Far Western blot for fH binding in the presence of antibodies. (B) Survival data in opsonophagocytic assay using anti-PspC var.-II IgG from rabbit2 immunized with PspC var.-II. Anti-PspC var.-II IgG recognizes PspC var.-III, in addition to its cognate target, PspC var.-II. Download FIG S2, PDF file, 0.1 MB. Copyright © 2018 Georgieva et al. 2018 Georgieva et al. This content is distributed under the terms of the Creative Commons Attribution 4.0 International license.

## DISCUSSION

Anti-PspC immunity is a major component of the total antiprotein antibody response during infection with *S. pneumoniae*. However, the impact of sequence diversity within *pspC* on the protein’s function or on the effectiveness of anti-PspC antibody responses has been variable in the few studies that have assessed it. In this study, we sought to address these issues and characterize the functional and immune implications of *pspC* diversity using antibodies to recombinant partial *pspC* gene products and isogenic strain sets that differ only at the *pspC* locus.

Despite extensive sequence variability, PspC contains several conserved regions and some regions of homology to PspA, another *S. pneumoniae* choline binding protein (6, 26, 31). To generate variant-specific antibodies, we produced recombinant truncated PspC protein fragments that possess only the unique sequence features that represent each PspC variant (Fig. 1; see also Text S1 and Fig. S3 in the supplemental material). In doing this, we were able to generate variant-specific instances of anti-PspC IgG that recognize their cognate target but not other PspC variants (Fig. 2). Indeed, we hypothesize that this approach may explain the greater specificity, both in recognition and in activity, observed in our study than in previous studies.

10.1128/mBio.00264-18.4 FIG S3  Alignment of protein sequences corresponding to truncated PspC variants I to IV. Download FIG S3, PDF file, 0.5 MB. Copyright © 2018 Georgieva et al. 2018 Georgieva et al. This content is distributed under the terms of the Creative Commons Attribution 4.0 International license.

Biochemical data on the structural landscape of PspC-fH and PspC-sIgA interactions suggest that the fH and sIgA binding domains on PspC are surface exposed and, therefore, potential antibody targets *in vivo*. This motivated us to investigate whether anti-PspC antibodies recognize the fH/sIgA binding domains and whether they impede PspC-fH and PspC-sIgA interactions in a variant-specific fashion. A previous study found little evidence of allelic specificity with respect to the ability of anti-PspC antibodies to inhibit binding of these host proteins to lysates of isolates expressing homologous variants. In particular, one antiserum (to variant 3 in that study) showed limited inhibition of binding even to the homologous variant, while another (variant 5) showed inhibition of binding to the homologous and several heterologous variants (35). We found that anti-PspC var.-I antibody prevented association of PspC var.-I with fH but not with PspC var.-II or var.-III (Fig. 3A). Similarly, anti-PspC var.-II antibody prevented association of PspC var.-II with fH but not with PspC var.-I or var.-III (Fig. 3A). Together, these findings point to a variant-specific function for antibodies in blocking fH binding. Our strategy of immunizing with the most variant-specific regions of the protein (as mentioned above) may underlie the greater specificity observed here than in previous work. A different choice of variants may also contribute to this finding. Notably, we found variability in anti-PspC var.-II IgG specificity depending on the source of antisera. Anti-PspC var.-II IgG from a second rabbit immunized with PspC var.-II showed variable specificity in blocking fH binding (Fig. S2A). Similarly, in an opsonophagocytic killing assay, anti-PspC IgG from the same rabbit showed recognition of PspC var.-III in addition to its cognate target, PspC var.-II (Fig. S2B). These findings underscore the variability inherent in biological systems and highlight the importance of maintaining awareness about differences in immune responses driven by host-intrinsic factors. Interestingly, while anti-PspC antibodies prevented association between PspC and fH, they did not affect interaction between PspC and sIgA (Fig. 3B). This is consistent with other findings showing that the fH and sIgA binding domains on PspC are distinct (38).

It is important to discuss our findings in the context of other studies on anti-PspC antibodies. Data from PspC epitope mapping studies exclude the fH domain from the list of epitopes within PspC that are recognized by serum samples from healthy adult humans (45). The physical association between PspC and fH *in vivo* is often invoked to explain these results. It is hypothesized that because the fH binding domain is occupied, it is not seen by the naive B cells that would recognize it if it were exposed. Furthermore, it has been suggested that, due to the species-specific interaction between PspC and fH, antibodies against the fH binding domain occur only in animal models where PspC does not interact with fH (42). However, it is likely that there exists an on-off rate for PspC-fH binding such that fH is not bound to PspC at all times. Furthermore, we found evidence for interactions between PspC and rabbit fH concurrent with our finding for the presence of anti-fH domain antibodies (data not shown). At this time, it remains unclear whether this is an outcome of the particular animal model that we utilized, and dissecting the mechanistic basis for this observation is beyond the scope of the current study. Instead, we highlight the potential biological relevance of anti-fH domain antibodies. Factor H acts to dampen the alternative complement pathway, and fH recruitment to the bacterial surface is a mechanism that *S. pneumoniae* employs to modulate host immune activation. Therefore, antibodies with the capacity to prevent association between PspC and fH would likely have an important biological role. Our findings could inform future vaccine design and underscore the utility of antibodies with specificity against the fH binding domain.

Our data fit within the larger context of investigations on the functional implications of PspC protein diversity (43). Here, we offer additional insight into the functional range of previously uncharacterized PspC protein variants. To measure the functional consequences of sequence diversity, we set out to evaluate whether our PspC variants differ in the capacity to bind human fH (Fig. 4). Because *pspC* expression is controlled, it is a possibility that any differences in fH binding may be an attribute of differential gene regulation (20, 46). Additionally, it was previously demonstrated that capsule modulates the amount of fH deposited on the bacterial surface (47, 48). While these are certainly important aspects of PspC biology, our goal was to evaluate the implications of sequence diversity alone for the functional range of PspC variants, excluding any contributions of genetic background and gene regulation mechanisms. Therefore, we designed isogenic *S. pneumoniae* strains that differ solely in the unique full-length PspC variant that they express. Our results show that these strains bind various amounts of fH and suggest that sequence diversity within *pspC* reflects functional differences (Fig. 4). There was little difference in fH binding results between *S. pneumoniae* strains bearing PspC var.-I, var.-II, or var.-IV. Moreover, a comparison with *S. pneumoniae* ΔPspC suggests that, in these strains, fH binding was essentially attributable to bacterial factors other than PspC. It remains possible that in strains bearing these PspC variants, genetic background plays a crucial role in regulation of PspC gene/protein levels and thereby effectively promotes fH binding. In contrast, *S. pneumoniae* PspC var.-III stood out with the highest fH binding capacity. Overall, our data add to our understanding of the functional implications of sequence diversity in PspC. The range in fH binding may be a reflection of functional constraints imposed on the PspC protein. Low levels of constraint would explain how PspC accommodates such extensive sequence diversity across a wide range of protein variants. Alternatively, the range of fH binding could reflect differences in immune responses between hosts (49). This would be consistent with a model where variability in human serum fH levels introduces selection with respect to *S. pneumoniae* to produce PspC variants with various fH binding capacities (49, 50).

Having produced variant-specific anti-PspC antibodies, we wanted to ask questions about the benefit of sequence diversity in the context of variant-specific immunity. We performed antibody-dependent opsonophagocytic assays and found that *S. pneumoniae* expressing PspC var.-II matching the antibody specificity was killed efficiently. Importantly, killing efficacy was not evident against *S. pneumoniae* expressing a mismatched PspC variant. Our data suggest that antigenic variation within the PspC pneumococcal antigen promotes immune evasion and confers a fitness benefit in the presence of variant-specific immunity. Our observations are consistent with previously reported data showing that antibodies raised against a particular PspC variant promote neutrophil-mediated killing of a *S. pneumoniae* strain expressing a matched PspC variant (36). Our results extend those findings in two ways. First, we show that PspC mismatch promotes survival, while the prior report showed that it reduced phagocytic uptake but did not compare levels of killing of matched versus mismatched PspC alleles. Second, our studies were performed in otherwise-isogenic strains, thereby isolating the impact of PspC allelic variation as opposed to any other differences between the isolates employed in the earlier study. Under the conditions employed in our experiments, anti-PspC var.-I and var.-IV antibodies seemed nonfunctional in opsonophagocytic killing assays. It remains possible that these antibodies have an alternative function *in vivo*.

It is important to discuss our findings in the context of natural immunity, which is multifaceted and likely characterized by variant-specific as well as cross-reactive antibody responses. Dissecting the relative contributions of all of these is beyond the scope of the current study. Instead, here, we address a conceptual issue about the relevance of sequence diversity in a setting of variant-specific immunity. We highlight findings of strongly variant-specific antibody activity (here, inhibition of fH binding and opsonophagocytic activity) against a pneumococcal protein antigen. This provides evidence that sequence diversity can provide an immune escape benefit in the presence of variant-specific antibody responses. We acknowledge that, in the context of natural infection, full-length antigenic variants bearing unique as well as cross-reactive epitopes would likely generate a multifaceted antibody response marked by variable specificity. Further studies investigating how this balance between specificity and cross-reactivity is biologically meaningful are warranted and will be crucial in advancing our understanding of the interplay between sequence diversity in protein antigens and host antibody immunity.

## MATERIALS AND METHODS

### Cloning of recombinant PspC proteins for expression in *Escherichia coli.*

PspC var.-I, var.-II, var.-III, and var.-IV were amplified from genomic DNA of the corresponding *S. pneumoniae* parent strain using the following primers: PspC var.-I forward primer 5’-GGAATTCAGCATATGAGTGGGGATACCCCCAAG-3’ and PspC var.-I reverse primer 5’-CCTTAAGTGGCGGCCGCTGGTTTTGGAGCTGGAGCTGG-3’; PspC var.-II forward primer 5′-GGAATTCAGCATATGAAGAATAACCTCACGGTT-3′ and PspC var.-II reverse primer 5′-CCTTAAGTGGCGGCCGCCTCTGGGTTTTCCGGCTGTGG-3′; PspC var.-III forward primer 5′-GGAATTCAGCATATGAACGAGGGAACTACCCAAGCA-3′ and PspC var.-III reverse primer 5′-CCTTAAGTGGCGGCCGCTGGATTTTCCGGCTGCGGTTT-3′; and PspC var.-IV forward primer 5′-GGAATTCAGCATATGAACGAGGGAAGTACCCAAGCA-3′ and PspC var.-IV reverse primer 5′-CCTTAAGTGGCGGCCGCTTCCGGCTCTGGTTTAACCTC-3′. Each of these constructs was then separately ligated into pET-21b (EMD Chemicals) using the restriction sites EcoRI and NotI, yielding a construct carrying an in-frame polyhistidine affinity tag (6×His tag) at the C terminus.

### Expression and purification of recombinant PspC proteins in *E. coli.*

Plasmids pET21bPspCVar-I, pET21bPspCVar-II, pET21bPspCVar-III, and pET21bPspCVar-IV were transformed into *E. coli* Rosetta (DE3) (Invitrogen) for protein expression. Luria-Bertani (LB) broth (1 liter) containing 100 µg/ml ampicillin was inoculated with 5 ml of overnight culture and incubated at 37°C to an optical density at 600 (OD_600_) of 0.6 to 1.0. The cells were cooled to room temperature for 15 to 30 min, after which 1 mM IPTG (isopropyl β-d-thiogalactopyranoside) was added and the cells were allowed to incubate overnight at 25°C. The cells were then centrifuged at 6,500 rpm for 1 h. The pellet was resuspended in buffer (300 mM NaCl, 50 mM Tris-HCl [pH 8.0], 10 mM MgCl_2_, protease inhibitor cocktail, 1 µg/ml DNase), sonicated, and centrifuged at 13,000 rpm for 30 min at 4°C. The supernatant was incubated with Ni^2+^-charged beads and then applied to a gravity column at 4°C. The cell lysate in the gravity column was washed first with buffer A (300 mM NaCl, 50 mM Tris-HCl [pH 8.0]) and then twice with buffer A plus 20 mM imidazole. The protein was eluted with 300 mM imidazole in buffer A and dialyzed overnight in 50 mM Tris-HCl (pH 8.0). The dialyzed protein was then purified by the use of a size exclusion column, concentrated, divided into aliquots, and stored at −80°C. Proteins were subjected to SDS-PAGE and visualized by staining with 0.05% Coomassie blue R-250. The concentrations of purified proteins were determined by the use of a Qubit protein assay kit.

### *In silico* characterization of *pspC* sequences.

The four *pspC* variant sequences in this study were previously identified within a collection of 616 asymptomatically carried *S. pneumoniae* isolates (5). Geneious software was used to analyze each *pspC* sequence. To identify regions of homology between the *pspC* genes as well as between PspC and PspA, we performed alignments and selected regions with low within-pair sequence identity for subsequent analysis.

### Production of isogenic strains.

Four PspC isogenic variants in the TIGR4 and 603 genetic background were constructed. To generate the TIGR4:PspC var.-I, TIGR4:PspC var.-II, TIGR4:PspC var.-III, and TIGR4:PspC var.-IV strains, the PspC locus in the TIGR4 and 603 strains were replaced with a SweetJanus cassette by using a previously described transformation protocol (51). The SweetJanus cassette was then replaced by the allele corresponding to the sequence of PspC var.-I, PspC var.-II, PspC var.-III, or PspC var.-IV to generate the variant strains.

### Preparation of protein extracts from *S. pneumoniae* strains.

Each *S. pneumoniae* strain was grown on solid medium (Trypticase soy agar with 5% sheep blood) for ~15 h at 37°C. Then, pneumococcal cells were harvested and resuspended in 1 ml 1× phosphate-buffered saline (PBS) buffer. Cells were then centrifuged at 4,000 × *g* for 10 min at room temperature, and the pellet was resuspended in 360 µl of lysis buffer with the following composition: 20 mM Tris-HCl, 2 mM EDTA, 1.2% Triton-X, and 20 µg/µl lysozyme. Samples were then incubated at 37°C for 90 min, subjected to vortex mixing for 1 min, and incubated at 55°C for 1 h. Following incubation, the lysate was centrifuged at 11,000 rpm for 30 min, and the supernatant (protein fraction) was removed for quantification using a Qubit protein assay kit. These protein fractions were subsequently used for Western blotting.

### Production of rabbit antisera.

To produce custom variant-specific antisera, rabbits were inoculated with 100 μg recombinant PspC var.-I, var.-II, var.-III, or var.-IV in complete Freund’s adjuvant. At days 14, 21, 49, and 77, rabbits received a boost of 50 μg recombinant PspC in incomplete Freund’s adjuvant. Production bleeds collected at day 84 were used to isolate the anti-PspC IgG component. These immunizations were performed by Cocalico Biologicals, Inc. (Stevens, PA) per established protocols.

### Western blotting.

Protein samples (10 µg per lane) were prepared in 4× SDS-PAGE loading dye, boiled for 10 min, separated on NuPAGE 10% bis-Tris gels, and transferred onto nitrocellulose membranes (Thermo Scientific). Membranes were blocked in TBST (150 mM NaCl, 25 mM Tris-HCl [pH 7.0], 0.1% Tween 20) containing 5% Blotto (Santa Cruz Biotechnology) for 1 h at room temperature and probed with antisera overnight at 4°C. Rabbit polyclonal anti-PspC var.-I, anti-PspC var.-II, anti-PspC var.-III, or anti-PspC var.-IV antisera were used at a 1:1,000 dilution in 1% Blotto. Membranes were washed in TBST and incubated for 1 h at room temperature with goat anti-rabbit IgG peroxidase-conjugated secondary antibody (Rockland). Blots were developed using a WesternSure ECL chemiluminescent substrate kit (Li-Cor) and visualized using a Bio-Rad ChemiDoc MP imaging system.

For Far-Western blots, recombinant PspC protein samples (5 µg per lane) were prepared in 4× SDS-PAGE loading dye, boiled for 10 min, separated on NuPAGE 10% bis-Tris gels, and transferred onto nitrocellulose membranes (Thermo Scientific). Membranes were blocked in TBST (150 mM NaCl, 25 mM Tris-HCl [pH 7.0], 0.1% Tween 20) containing 5% Blotto (Santa Cruz Biotechnology) for 1 h at room temperature and incubated with recombinant human fH (Quidel) or human sIgA purified from colostrum (Accurate Chemical) overnight at 4°C. The membranes were then washed and probed with murine anti-human factor H (Quidel) (clone A229) or murine anti-sIgA (Sigma-Aldrich) (clone GA-1). Antibodies were used at a 1:1,000 dilution in 1% Blotto. Finally, membranes were washed in TBST and incubated for 1 h at room temperature with goat anti-mouse IgG peroxidase-conjugated secondary antibody (Rockland). Blots were developed using a WesternSure ECL chemiluminescent substrate kit (Li-Cor) and visualized using a Bio-Rad ChemiDoc MP imaging system. To test for inhibition of fH or sIgA binding, prior to incubation with recombinant fH and sIgA, membranes were incubated with rabbit antisera against PspC var.-I, anti-PspC var.-II, anti-PspC var.-III, or anti-PspC var.-IV at a 1:1,000 dilution in 1% Blotto overnight.

### Flow cytometry.

Overnight *S. pneumoniae* (TIGR4 isogenic strains) cultures were used to inoculate liquid THY cultures. Cultures were grown to an OD_620_ of 0.2 and then washed twice in 1× PBS. Approximately 10 × 10^6^ cells were collected for staining. For fH binding to whole bacteria, *S. pneumoniae* cells were incubated with IgG-depleted normal human serum (Quidel) for 1 h at 37°C. Following incubation, cells were washed twice in PBS containing 2% fetal bovine serum (FBS) and incubated with goat antisera against human factor H (Quidel) for 30 min at 37°C. Cells were then incubated with Alexa Fluor 488-conjugated anti-goat IgG (Thermo Fisher) (clone A11055). For binding of anti-PspC rabbit antibodies to whole bacteria, ~10 × 10^6^ *S. pneumoniae* cells were incubated with purified anti-PspC IgG for 30 min at 37°C. Afterward, cells were washed twice in PBS containing 2% FBS and stained with goat anti-rabbit IgG FITC-conjugated antibody (Abcam, Inc.) (clone ab6717). Cells were incubated at 4°C for 30 min and then washed with PBS containing 2% FBS. Finally, the cells were fixed in fixation buffer (data were immediately acquired using an LSR flow cytometer [BD Biosciences]). Data were analyzed with FlowJo software (Tree Star, San Carlos, CA).

### Isolation of immunoglobulin G (IgG).

To isolate IgG from rabbit anti-PspC antisera, 1 ml of rabbit antisera was mixed with 4 ml of binding buffer (0.1 M NaH_2_PO_4_ [pH 8.0]). The sample was then applied to a prewashed protein G GraviTrap column (GE Healthcare) and incubated on column for 10 min at room temperature. The column was washed three times with 10 ml binding buffer, and IgG was eluted with 4 ml 0.1 M glycine (pH 2.5). To neutralize, 200 µl Tris (pH 8.5) was added per milliliter of eluate.

### IgG depletion.

To deplete IgG from normal human serum, 1 ml of rabbit antisera was mixed with 4 ml of binding buffer (0.1 M NaH_2_PO_4_ [pH 8.0]). The sample was then applied to a prewashed protein G GraviTrap column (GE Healthcare) and incubated on the column for 10 min at room temperature. Column flowthrough containing the IgG-depleted fraction was collected.

### Opsonophagocytic assay with differentiated HL-60 cells.

The HL-60 tissue culture human cell line (promyelocytic leukemia cells [American Type Culture Collection; catalog no. CCL-240]) was used as a source of effector cells. HL-60 cells were maintained in Iscove’s modified Dulbecco’s medium (IMDM) (Corning; catalog no. 10-016-CV) containing 2 mM l-glutamine and 25 mM HEPES and supplemented with 20% fetal bovine serum (American Type Culture Collection; catalog no. 30-2020). Cells were grown in suspension to ≤1 × 10^6^ cells/ml at 37°C in a 5% CO_2_ atmosphere. Differentiation was carried out in supplemented IMDM (as described above) containing 0.8% *N*,*N*-dimethylformamide (Sigma; catalog no. D4551). Flasks to be differentiated were inoculated at 5 × 10^5^ cells/ml as judged by viable counts with trypan blue exclusion. Cultures were incubated for 5 days, and the differentiation medium was not replaced during this time. For the functional assay, the bacteria (*S. pneumoniae* 603 isogenic strains), differentiated HL-60 cells, serum, and complement source were prepared as described below. For preparation of the serum, each serum sample was diluted in opsonophagocytosis buffer for a total of 5 dilutions. Once all serum samples were prepared, 80 µl of bacterial suspension that had been appropriately diluted (~1,000 CFU) was added to each well. Following a 30-min incubation at 37°C, 10 µl complement (baby rabbit complement [Pel-Freez; catalog no. 31064]) was added to each well. The assay plate was allowed to incubate for another 30 min at 37°C. Immediately after this, differentiated HL-60 cells were added to each well. Differentiated HL-60 cells were used in the opsonophagocytic assay at an effector/target cell ratio of 400/1. Differentiated cells were harvested by centrifugation (1,700 rpm) for 10 min at room temperature. The cell pellet was washed twice in Hanks’ buffer without Ca^2+^ and Mg^2+^ (Corning; catalog no. 21-022-CV). Finally, the cells were resuspended to 1 × 10^7^ cells/ml in Hanks’ buffer with Ca^2+^ and Mg^2+^ (Corning; catalog no. 21-023-CV) supplemented with 0.1% gelatin. The total number of HL-60 cells to be added per well was 4 × 10^5^ in a 40-µl volume. The assay plate was incubated at 37°C for 45 min with horizontal shaking (700 rpm) to promote the phagocytic process. All samples were run in triplicate. An aliquot from each well was plated onto solid medium (Trypticase soy agar with 5% sheep blood) to enumerate viable CFU after overnight incubation. A count of the initial number of viable bacteria added per well at time zero was included in each run. For each sample, the CFU counts at time point 45 min were divided by the CFU counts at time point 0 to determine the percentage of CFU that survived.
