# Supplementary material for: Antigenic Variation in Streptococcus pneumoniae PspC Promotes Immune Escape in the Presence of Variant-Specific Immunity
Source: mBio. 2018 Mar 13;9(2):e00264-18. doi: 10.1128/mBio.00264-18 (PMC5850329; doi:10.1128/mBio.00264-18)
Supplement: TEXT S1 [file mbo002183775s1.pdf]

Supplementary Information. Sequences of full length PspC variants and truncated protein fragments (in grey).

PspC Var-I

```
1        10        20        30        40        50
|        |         |         |         |         |
ATGTTTGCATCAAAAAGCGAAAGAAAAGTACATTATTCAATTCGTAAATT
TAGTGTTGGAGTAGCTAGTGTAGTAGTTGCTAGCTTGTTCTTAGGAGGAG
TAGTCCATGCAGAAGGGGTTAGAAGTGGGGATACCCCCAAGGTTACATCT
AGTGGGCAAGATATATCGAAGAAGTATGCTGATGAAGTCAAGTCGCATCT
AGAAAAAATATTGAGTGAGATCCAATTAGATAAAAGAAAACATACCCAAA
ATCTCGCCTTCAACAAAAAGTTGAGCAGAATTCAAACGGAGTATTTCTAT
TTAAAAAAGAAGTTGAAAGCTGAGTTGACGTCAAAAACAAAAGAAGAGTT
AACGTCAAAAACAGAAGAGTTGACGTCAAAAACAAAAAAAGAGTTAGACG
CAGCTTTTGAGCAGTTTAAAAAAGATACATTATCAACAGAACCAGAAAAA
AAGGTTGCAGAAGCTCAGAAGAAGGTAGCAGAAGCTAAGAAAAAAGCCGA
GGATCAAAAAGAAGAAGATCGTCGTAACTACCCAACCAATACTTACAAAA
CGCTTGAACTTGAAATTGCTGAGTTCGATGTGAAAGTTAAAGAAGCGGAG
CTTGAACTATTAAAAGAGAAAGCTAAGGAATCTCGAGACGAGGAAAAAAT
TAAGCAAGCAGAAGCGGAAGTTGAGAGTAAACAAGCTGAGGCTACAAGGT
TAGAAAACATCAAGACAGATCGTAAAAAAGCAGAAGAAGAAGCTAAACGA
AAAGCAGATGCTAAGTTGAAGGAAGCTAATGTAGCGACTTCAGATCAAGG
TAAACCAAAGGGGCGGGCAAAACGAGGAGTTCCTGGAGAGCTAGCAACAC
CTGATAAAAAAGAAAATGATGCGAAGTCTTCAGATTCTAGCGTAGGTGAA
GAAACTCTTCCAAGCTCATCCCTGAAATCAGGAAAAAAGGTAGCAGAAGC
TGAGAAGAAGGTTGAAGAAGCTGAGAAAAAAGCCAAGGATCAAAAAGAAG
AAGATCGCCGTAACTACCCAACCAATACTTACAAAACGCTTGAACTTGAA
ATTGCTGAGTCCGATGTGAAAGTTAAAGAAGCGGAGCTTGAACTAGTAAA
AGAGGAAGCTAAGGAACCTCGAGACGAGGAAAAAATTAAGCAAGCAAAAG
CGGAAGTTGAGAGTAAACAAGCTGAGGCTACAAGGTTAGAAAAAATCAAG
ACAGATCGTAAAAAAGCAGAAGAAGAAGCTAAACGAAAAGCAGCAGAAGA
AGATAAAGTTAAAGAAAAACCAGCTGAACAACCACAACCAGCGCCGGCTC
CTCAACCAGAAAAACCAGCTCCAAAACCAGAAAAACCAGCTCCAGCTCCA
AAACCAGAGAATCCAGCTGAACAACCAAAAGCAGAAAAACCAGCTGATCA
ACAAGCTGAAGAAGACTATGCTCGTAGATCAGAAGAAGAATATAATCGCT
TGACTCAACAGCAACCGCCAAAAACTGAAAAACCAGCACAACCATCTACT
CCAAAAACAGGCTGGAAACAAGAAAACGGTATGTGGTACTTCTACAATAC
TGATGGTTCAATGGCGACAGGATGGCTCCAAAACAATGGCTCATGGTACT
ACCTCAACAGCAATGGCGCTATGGCGACAGGATGGCTCCAAAACAATGGT
TCATGGTACTATCTAAACGCTAATGGTTCAATGGCAACAGGATGGCTCCA
AAACAATGGTTCATGGTACTACCTCAACGCTAATGGTTCAATGGCGACAG
GATGGCTCCAATACAATGGTTCATGGTACTACCTCAACGCTAATGGTGAT
ATGGCGACAGGATGGCTCCAAAACAATGGCTCATGGTACTACCTAAACGC
TAATGGTGATATGGCGACAGGATGGCTCCAAAACAACGGTTCATGGTACT
ACCTAAACAGCAATGGTGCTATGGTAACAGGATGGCTCCAAAACAATGGC
TCATGGTATTACCTCAACGCTAATGGTGATATGGCGACAGGTTGGGTGAA
AGATGGAGACACCTGGTACTATCTTGAAGCATCAGGTGCTATGAAAGCAA
GCCAATGGTTCAAAGTATCAGATAAATGGTACTATGTCAATGGCTCAGGT
GCCCTTGCAGTCAACACAACTGTAGATGGCTATGGAGTCAATGCCAATGG
TGAATGGGTAAAC
```

PspC Var-II

```
1        10        20        30        40        50
|        |         |         |         |         |
ATGTTTGCATCAAAAAGCGAAAGAAAAGTACATTATTCAATTCGTAAATT
TAGTATTGGAGTAGCTAGTGTAGTAGTTGCCAGCTTGTTCTTAGGAGGAG
TAGTCCATGCAGAAGGGGTTAGGAGTAAGAATAACCTCACGGTTACATCT
```

AGTGGGCAAGATATATCGAAGAAGTATGCTGATGAAGTCGAGTCGCATCT
ACAAAGTATATTGAAGGATGTCAATAAAAATTTGAAAAAAGTTCAACATA
CCCAAAATGTCGACTTCAACAAAAAGTTGAGCAGAATTAAAACGAAGTAT
TTGTATGGATTAAAAGAGAAGTCGGAAGCTGAGTTGACGTTAAAAACAAA
AGAAACAAAAGAAGAGTTAACCGCAGCTTTTGAGCAGTTTAAAAAAGATA
CATTGAAATCAGGAAAAAAGGTAGCAGAAGCTGAGAAAAAAGCCAAAGCT
CAAAAAGAAGAAGATCGCCGTAACTACCCAACCAATACTTACAAAACAAT
CGAACTGGAAATTGCGGAAGCAGAAGTAGGGGTCGCCAAGGCAGAGCTTG
AGCTTGAGTTTGCACAAGCTCAAGTTCAAATACCTCAAGATACTGAGAAA
ATTAATGCTGCTAAATCTAAAGTAGAAGCTGCTAAAAGTAATGTTAAAAA
ACTAGAAAAAATTAAATCAGATATTGAAAAAACGTATTTGTATAAATTAG
ATAACTCAACCAAAGAAACGCCAAAACCTAGAGTGCGAAGAAATTCTCCG
GAAATAAAAGCTAAGGGCAGAGTCAAAAATTATGAAGAGGCTAATATTGA
ACTTTCTAAATATATGACTGATTTGTATAAATTAGATAACTCAACCAAAG
AAACGCCAAAATCTAGAGTGCGAAGAAATTCTCCGCAAGTAGGCGATTCG
AGAGAACTTAAGGAAACGATAGACAAAGCGAAAGAAACTCTGTCTACCTA
TATGGTAACTCGTTTAACGAAGCTGGATCCATCTGTTTTTTGGTTTGCAG
ATCTTCTTATGGATGCTAAGAAGGTTGTGGAAGAATACAAGACAAAATTA
GAGGATGCTTCAGATAAAAAATCGGTAGAAGACTTGCGAAAGGAAGCAGA
AGGAAAAATAGAGTCTCTTATCGTGACTCACCAAAATAGAGAAAAAGAAA
ACCAACCAGCACCCCAACCAGGAGGACAAGCAGGTGGTTCAATGGTTGTA
CCACCGGTGACGCAAACACCTCCATCAACTTCCCAAAGTCCAGGACAAAA
GGCGACCGAAGCTGAAAAGAAAAAGTTACAAGACTTGATTCGTCAATTCC
AAGAAGCCTTGAACAAACTAGACGATGAAACAAAGACTGTTCCAGATGGG
GCTAAACTCACAGGAGAAGCTGGAAAAGCCTATAATGAGACTAGAACTTA
TGCGAAAGAAGTTGTTGACAAAAGCAAGAAGCTTCTATCACAGACAGCAG
TGACAATGGATGAATTGGCAATGCAATTAACCAAATTGAACGATGCCATG
TCTAAATTGAAAGAAGCTAAAGCGAAATTGGTACCAGAGGTTAAACCACA
GCCGGAAAACCCAGAGCCAAAACCACAACCAGAGGGTGAGAAACCAAGCG
TACCAGATATTAATCAGGAGAAAGAAAAAGCTAAACTTGCTATAGCAACA
TACATGAGCAAGATTTTAGATGATATAAAGAAACATCATCTGAAGAAAGA
AAAACATCATCAGATTGTTGCTCTTATTAAGGACCTTGATAAACTTAAAA
AGCAAGCACTTTCTGAAATTGATAATGTAAATACCAAAGTAGAAATTGAG
AATACAGTCCACAAGGTATTTGCAGACATGGATACGGTTGTTACTAAATT
CCAAAAAGGCTTAATTCAGAACACACCGCAGGTTCCAGAAGCACCAAAGA
GCCCAGAGGTACCAAAGGTTCCAGATACACCAAAGGCTCCAGACACACCG
CAAGTTCCGGAAGCACCAAAGAGCCCAGAGGTACCAAAGGTTCCAGATAC
ACCAAAGGCTCCAGACACACCGCAAGTTCCGGAAGCACCAAAGAGCCCAG
AGGTACCAAAGGTTCCAGATACACCAAAGGCTCCGGACACACCGCAAGTT
CCAGAAGCACCAAAGAGCCCAGAGGTACCAAAGGTTCCAGATACACCAAA
GGCTCCGGACACACCGCAGGTTCCAGAAGCACCAAAGNACATGGATACGG
TTGTTACTAAATTCCAAAAAGGCTTAATTCAGAACACACCGCAGGTTCCA
GAAGCACCAAAGAGCCCAGAGGTACCAAAGGTTTCAGATACACCAAAGGC
TCCGGACACACCGCAGGTTCCAGAAGCACCAAAGAGCCCAGAGGTACCAA
AGGTTCCAGAAGCACCAAAGGCTCCAGACACACCGCAAGTTCCGGAAGCA
CCAAAGAGCCCAGAGGTACCAAAGGTTCCAGATACACCAAAGGCTCCAGA
CACACCGCAAGTTCCGGAAGCACCAAAGAGCCCAGAGGTACCAAAGGTTC
CAGATACACCAAAGGCTCCGGACACACCGCAGGTTCCAGAAGCACCAAAG
GCTCCAGACACACCGCAAATTCCGGAAGCACCAGCTCCAGAAACTCCGGC
TCCAGCTCCAGAAGCTCCAAAAACAGGCTGGAAACAAGAAAACGGTATGT
GGTACTTCTACAATACTGATGGTTCAATGGCAACAGGCTGGCTAGAATAC
AATGGCTCATGGTACTATCTCAACGCTAATGGTGCTATGGCAACAGGTTG
GCTAGAATACAATGGTTCATGGTACTACCTCAATACTAATGGTGCTATGG
AGACAGGTTGGCTAGAATACAATGGTTCATGGTACTACCTCAATACTAAT
GGTGCTATGGAGACAGGTTGGCTAGAATACAATGGTTCATGGTACTACCT
NNNNNNNNNNNNNNNNNNNNNNNNNNNNNNNNNNNNNNNNNNNNNNNNNN
NNNNNNNNNNNNNNNNNNNNNNNNNNNNNNNNNNNNNNNNNNNNNNNNNN

NNNNNNNNNNNNNNNNNNNNNNNNNNNNNNNNNNNNNNNNNNNNNNNNNN
NNNNNNNNNNNNNNNNNNNNNNNNNNNNNNNNACAGGTTGGCTAGAATAC
AATGGTTCATGGTACTACCTCAATAC

PspC Var-III

```
1        10        20        30        40        50
|        |         |         |         |         |
ATGTTTAAATCAAATTATGAAAGAAAAATGTGCTATTCTATTCGAAAATT
TAGTATAGGAGTAGCTAGTGTAGCTGTTGCCAGTCTTGTTATGGGAAGTG
TGGTTCATGCGACAGAGAACGAGGGAACTACCCAAGCACCCACTTCTTCT
AATAGGGGAAATGAAAGTCAGGCAGAACAACGTAGAGAACTCGATTTAGA
ACGAGATAAGGTAAAGAAAGAGGTCAGGGAATATAAAGAAAAAAAAGTGA
AAGAGCTCTATTCAAAATCAACTAAAAGTCGACATAAGAAGACTGTAGAT
ATAGTTAACAAGTTGCAAAACATTAATAACGAGTATTTGAATAAAATAAT
TCAATCAACCTCAACATACGAAGAACTACAGAAACTGATGATGGAGAGTC
AATCAGAAGTAGATAAAGCTGTGTCTGAATTTGAAAAGGACTTATCTTCT
TCGTCAAGTTCAGGCTCTTCCACGGAACCGGAAGCTTCAGATACAGCGAA
GCCAAACAAGCCGACAGAACTAGAAAAAAAGGTAGCAGAAGCTCAGCAGA
AGGTTGAAGAAGCTGAGAAAAAAGCCAAGGATCAAAAAGAAGAAGATTAC
CGTAACTACCCAACCATTACTTACAAAACGCTTGAACTTGAAATTGCTGA
GTTCGATGTGAAAGTTAAAGAAGCGGAGCTTGAACTATTAAAAGTGAAAG
CTAAGGAATCTCGAGACGAGAAAAAAATTAAGCAAGCAGAAGCGGAAGTT
GAGAGTAAACAAGCTGAGGCTACAAGGTTAAAAAAAATCAAGACAGATCG
TAAAAAAGCAGAAGAAGAAGCTAAGTTGAAGGAAGCTGTTGAAAAGAATG
CAGCGACTTCAGAGCAAGGTAAACCAAAGAGGCGGGTAAAACGAGGAGCT
CTTGGAGAGCAAGCAACACCTGATAAAAAAGATTATTTTGAAAAAGACTT
CCGTCCAGCTTTCAATAAAAACCAGCAGATGGTAGCCATTCAAGAATCCT
TGAACAAACTAGATGATGAAACAAAAACTGTTCCAGATGGGGCTAAACTC
ACAGGAGAAGCTGGAAATGCCTATAATGAGGTCAGAGATTATGCAATAAA
AGTTGTTTCTGAAAACAAGAAACTTCTATCACAGACAGCAGTGACAATGG
ATGAACTGGCAATGCAATTAACCAAATTGAACGATGCCATGTCTAAATTG
AAAGAAGCTAAAGCGAAATTGGTACCAGAGGTTAAACCGCAGCCGGAAAA
TCCAGAGCATCAAAAACCAACAACTCCAGCTCCGGATACCAAACCAATCC
CTCAACCAGAGGGTAAGAAACCAAGCGTACCAGATATTAATCAGGAAAAA
GAAAAAGCTAAGCTTGCTGTAGCAACCTACATGAGCAAGATTTTAGATGA
TATACAAAAACATCATCTGCAGAAAGAAAAACATCGTCAGATTGTTGCTC
TTATTAAGGAGCTTGATGAGTTTAAAAAGCAAGCTCTTTCTGAAATTGAT
AATGTAAATACCAAAGTAGAAATTGAAAATACAGTCCACAAGATATTTGC
AGACATGGATGCAGTTGTGACTAAATTCAAAAAAGGCTTAACTCAGGACA
CACCAAAAGAACCAGATAACAAAAAGCCATCTGCTCCAAAACCAGGTATG
CAACCAAGTCCTCAACCAGAAGGCAAGAAACATAATAAAAACACCCCAAA
AGTTAGATTTTTTCTGTCTAACTTTTGGGGTGTAGTTCATTTTGAGCTTT
CCCGTTTTTCTGAGTTG
```

PspC Var-IV

```
1        10        20        30        40        50
|        |         |         |         |         |
ATGTTTGCATCAAAAAGCGAAAGAAAAGTACATTATTCAATTCGTAAATT
TAGTATTGGAGTAGCTAGTGTAGCTGTTGCCAGTCTTGTTATGGGAAGTG
TGGTTCATGCGACAGAGAACGAGGGAAGTACCCAAGCAGCCACTTTTTCT
AATATGGCAAATAAAAGTCAGACAGAACAAGGAGAAATCAATATAGAACG
AGATAAGGCAAAGACAGCGGTCAGTGAATATAAAGAAAAAAAAGTGAGTG
AGATCTATACAAAATTAGAGAGAGATAGACATAAAGATACTGTAGATCTA
GTTAACAAGTTGCAAGAGATTAAGAACGAGTATTTGAATAAAATAGTTGA
ATCAACCTCAAAAATCGAAATACAGGGACTTATTACAACAAGTCGATCAA
AACTAGATGAAGCTGTGTCTAAATATAAAAAGGCCCCATCTTCTTCGTCA
AGTTCAGGCTCCTCCACTAAACCAGAAACTCCGCAGCCGGAAACATCAAA
```

ACCAGAGGTTAAACCAGAGCCGGAAACACCAAAACCAGAGGTTAAACCAG
AGCCGGAAACACCAAAACCAGAGGTTAAACCAGAGCCGGAAACACCAAAA
CCAGAGGTTAAACCAGAGCCGGAAACACCAAAACCAGAGGTTAAACCAGA
GCCGGAAACACCAAAACCAGAGGTTAAACCAGAGCCGGAAACACCAAAAC
CAGAGGTTAAACCAGAGCCGGAAACACCAAAACCAGAGGTTAAACCAGAG
CCGGAAACACCAAAACCAGAGGTTAAACCAGAGCCGGAAACACCAAAACC
AGAGGTTAAACCAGAGCCGGAAACACCAAAACCAGAGGTTAAACCAGAGC
CGGAAACACCAAAACCAGAGGTTAAACCAGAGCCGGAAACACCAAAACCA
GAGGTTAAACCAGAGCCGGAAACACCAAAACCAGAGGTTAAACCAGAGCC
GGAAACACCAAAACCAGAGGTTAAACCAGAGCCGGAAACACCAAAACCAG
AGGTTAAACCAGAGCCGGAAACACCAAAACCAGAGGTTAAACCAGAGCCG
GAAACACCAAAACCAGAGGTTAAACCAGAGCCGGAAACACCAAAACCAGA
GGTTAAACCAGATAATAGCAAGCCACAAGCAGATGATAAGAAGCCATCAA
CTCCAAATAATTTAAGCAAGGACAAGCAATCTTCTAACCAAGCTTCAACA
AACGAAAACAAGAAGCAAGGTCCAGCAACAAATAAACCGAAGAAGTCATT
GCCATCAACTGGATCTATTTCA
