# Supplementary material for: Antigenic Variation in Streptococcus pneumoniae PspC Promotes Immune Escape in the Presence of Variant-Specific Immunity
Source: mBio. 2018 Mar 13;9(2):e00264-18. doi: 10.1128/mBio.00264-18 (PMC5850329; doi:10.1128/mBio.00264-18)
Supplement: FIG S1 [file mbo002183775sf1.pdf]

A

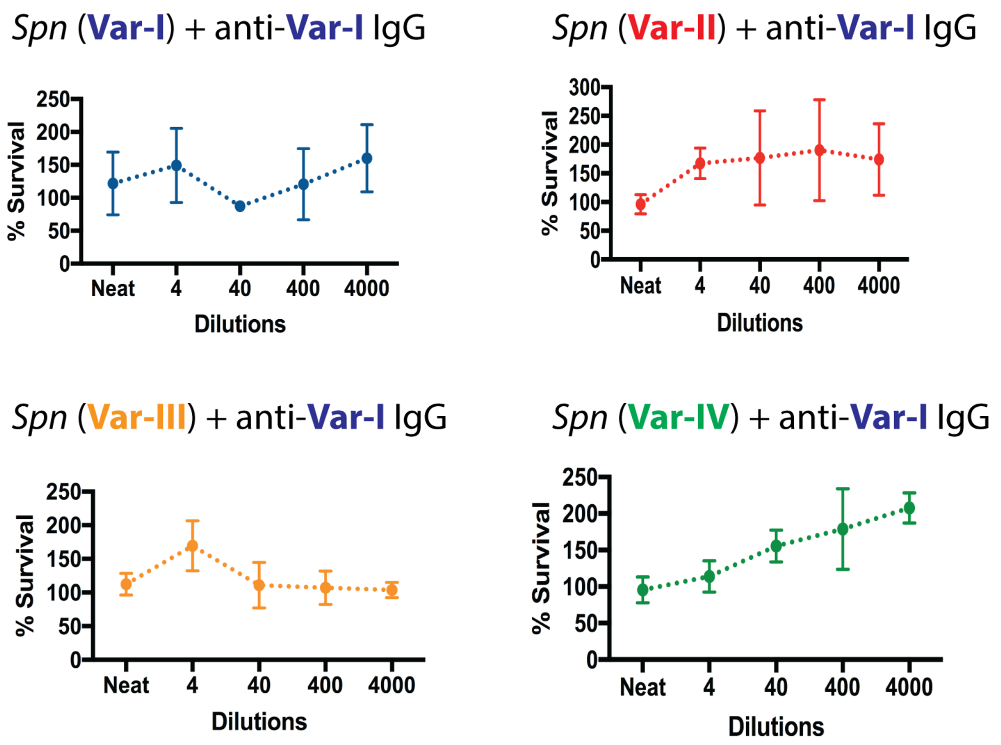

Spn (Var-I) + anti-Var-I IgG
Spn (Var-II) + anti-Var-I IgG
Spn (Var-III) + anti-Var-I IgG
Spn (Var-IV) + anti-Var-I IgG
% Survival
Dilutions
Neat
4
40
400
4000

B

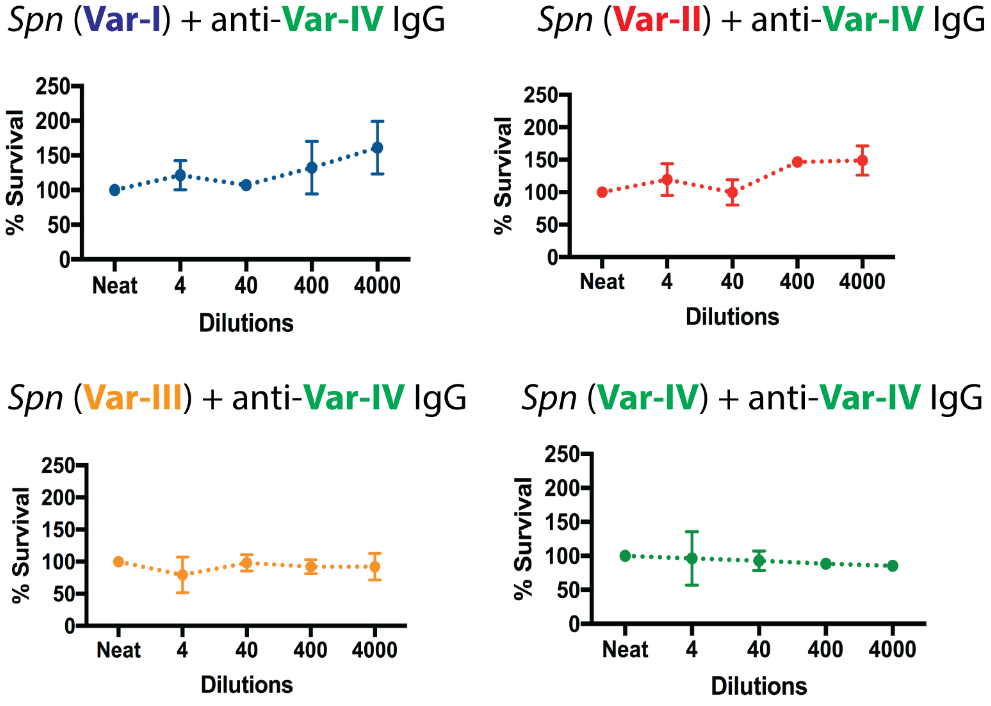

Spn (Var-I) + anti-Var-IV IgG
Spn (Var-II) + anti-Var-IV IgG
Spn (Var-III) + anti-Var-IV IgG
Spn (Var-IV) + anti-Var-IV IgG
% Survival
Dilutions
Neat
4
40
400
4000
