# Supplementary figures and images for: Antigenic Variation in Streptococcus pneumoniae PspC Promotes Immune Escape in the Presence of Variant-Specific Immunity
Source: mBio. 2018 Mar 13;9(2):e00264-18. doi: 10.1128/mBio.00264-18 (PMC5850329; doi:10.1128/mBio.00264-18)

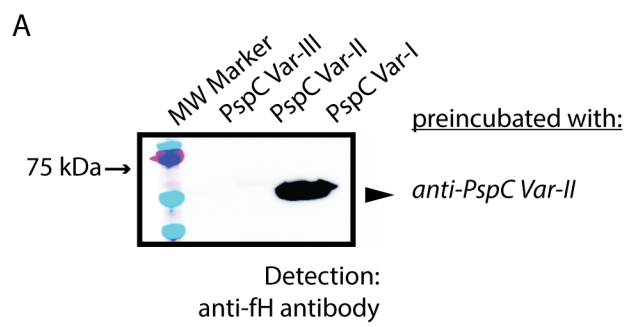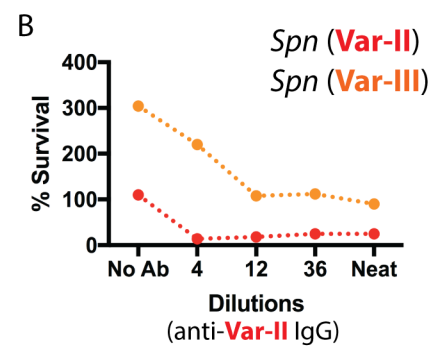

Supplement: FIG S2 [file mbo002183775sf2.pdf]
