# Supplementary material for: Antigenic Variation in Streptococcus pneumoniae PspC Promotes Immune Escape in the Presence of Variant-Specific Immunity
Source: mBio. 2018 Mar 13;9(2):e00264-18. doi: 10.1128/mBio.00264-18 (PMC5850329; doi:10.1128/mBio.00264-18)
Supplement: FIG S3 [file mbo002183775sf3.pdf]

```
            1         10        20        30        40        50        60
            |         |         |         |         |         |         |
PspC Var-I   MSGDTPKVTSSGQDISKKYADEVKSHLEKILSEI--QLDKRKHTQNLAFNKKLSRIQTEY
PspC Var-II  -MKNNLTVTSSGQDISKKYADEVESHLQSILKDVNKNLKKVQHTQNVDFNKKLSRIKTKY
PspC Var-III ------------------------------------------------------------
PspC Var-IV  ------------------------------------------------------------

PspC Var-I   FY-LKKKLKAELTSKTKEELTSKTEELTSKTKKELDAAFEQFKKDTLSTEPEKKVAEAQK
PspC Var-II  LYGLKEKSEAELTLKTKE------------TKEELTAAFEQFKKDTL---------KSGK
PspC Var-III ------------------------------------------------------------
PspC Var-IV  ------------------------------------------------------------

PspC Var-I   KVAEAKKKAEDQKEEDRRNYPTNTYKTLELEIAEFDVKVKEAELELLKEKAKESR---DE
PspC Var-II  KVAEAEKKAKAQKEEDRRNYPTNTYKTIELEIAEAEVGVAKAELELEFAQAQVQIPQ-DT
PspC Var-III --------------------MGTTQAPTSSNRGNESQAEQRRELDLERDKVKKEVREYKE
PspC Var-IV  ----------------------------------MKSQTEQGEINIERDKAKTAVSEYKE

PspC Var-I   EKIKQAEAEVE-SKQAEATRLENIKTDRKKAEEEAKRKADAKLKEANVATSDQGKPKGRA
PspC Var-II  EKINAAKSKVEAAK-SNVKKLEKIKSDIEKTYLYKLDNSTKETPKPRVRRNSPEIKAKGR
PspC Var-III KKVKELYSKSTKSRHKKTVDIVNKLQNINNEYLNKIIQSTSTYEELQKLMMESQSEVDKA
PspC Var-IV  KKVSEIYTKLERDRHKDTVDLVNKLQEIKNEYLNKIVESTSKI-EIQGLITTSRSKLDEA

PspC Var-I   KRGVPGELATPDKKENDAKSSDSSVGEETLPSSSLKSGKKVAEAEKKVEEAEKKAKDQKE
PspC Var-II  VKNYEEANIELSKYMTDLYKLDNSTKET--PKSRVRRNSPQVGDSRELKETIDKAKETLS
PspC Var-III VSEFEKDLSS-SSSSGSSTEPEAS--DTAKPNKPTELEKKVAEAQQKVEEAEKKAKDQKE
PspC Var-IV  VSKYKKAPSS-SSSSGSSTKPETPQPETSKPEVKPEPETPKPEVKPEPETPKPEVKPEPE

PspC Var-I   EDRRNYPTNTYKTLELEIAES-DVKVK-EAELELVKEEAKEPRDEEKIKQAKAEVESKQA
PspC Var-II  T----YMVTRLTKLDPSVFWFADLLMDAKKVVEEYKTKLEDASDKKSVEDLRKEAEGKIE
PspC Var-III EDYRNYPTITYKTLELEIAEF-DVKVK-EAELELLKVKAKESRDEKKIKQAEAEVESKQA
PspC Var-IV  T-----PKPEVKP-EPETPKP-EVKPEPETPKPEVKPEPETPKPEVKPEPETPKPEVKPE

PspC Var-I   EATRLEKIKTDRKKAEEEAKRKAAEEDKVKEKPAEQPQPAPAPQPEKPAPKPEKPAPAPK
PspC Var-II  S---LIVTHQNREKENQPAPQPGGQAGGSMVVPPVTQTPPS-TSQSPGQKATEAEKK---
PspC Var-III EATRLKKIKTDRKKAEEEAKLKEAVEKNAATSEQGKPKRRV-KRGALGEQATPDKKDYFE
PspC Var-IV  PETPKPEVKPEPETPKPEVKPEPETPKPEVKPEPETPKPEVKPEPETPKPEVKPEPETPK

PspC Var-I   P-----------------------------------------------------------
PspC Var-II  -------KLQDLIRQFQEALNKLDDETKTVPDGAKLTGEAGKAYNETRTYAKEVVDKSKK
PspC Var-III KDFRPAFNKNQQMVAIQESLNKLDDETKTVPDGAKLTGEAGNAYNEVRDYAIKVVSENKK
PspC Var-IV  PEVKPEPETPKPEVKPEPETPKPEVKP---------------------------------

PspC Var-I   ------------------------------------------------
PspC Var-II  LLSQTAVTMDELAMQLTKLNDAMSKLKEAKAKLVPEVKPQPENPE---
PspC Var-III LLSQTAVTMDELAMQLTKLNDAMSKLKEAKAKLVPEVKPQPENPEHQK
PspC Var-IV  ------------------------------------------------
```
